# *TNNT3* as a candidate node in breast cancer mechanobiology: current evidence, mechanistic models, and key knowledge gaps

**DOI:** 10.3389/fcell.2026.1836170

**Published:** 2026-05-15

**Authors:** Liyun Wei, Lemuge Qi, Xiaoqian Yu, Aiping Shi, Zhu Zhu

**Affiliations:** 1 The First Hospital of Jilin University, Changchun, Jilin, China; 2 Department of Breast Surgery, General Surgery Center, The First Hospital of Jilin University, Changchun, Jilin, China

**Keywords:** actomyosin contractility, breast cancer, mechanobiology, risk loci, TNNT3, tumor microenvironment

## Abstract

Breast cancer progression is increasingly recognized as a mechanically regulated process shaped by reciprocal interactions between tumor cells and the tumor microenvironment (TME). Mechanotransduction, cytoskeletal remodeling, cell–matrix adhesion, and extracellular matrix reorganization all contribute to tumor growth, invasion, and metastatic dissemination. TnT3, traditionally known as a component of the troponin (Tn) complex involved in striated muscle contraction, has recently attracted attention beyond its canonical role. In our previous study, we identified a significant genetic association between *TNNT3* and triple-negative breast cancer (TNBC) using Mendelian randomization (SMR) analysis, colocalization (Coloc) analysis, and integrated gene expression profiling. In this review, we distinguish limited direct evidence in breast cancer from broader indirect mechanobiological support and from explicitly speculative models. Rather than proposing *TNNT3* as a validated driver, we position it as a low-evidence but biologically interesting candidate whose relevance remains to be tested in spatially and cell-type-resolved systems.

## 1 Introduction

Breast cancer remains a major global public health problem, with a rising clinical and economic burden. In 2022, it was the most common cancer among women in 157 of 185 countries, with approximately 2.3 million new cases and 670,000 deaths worldwide (World Health Organization, 2017). Breast cancer incidence shows marked geographic and population heterogeneity and is generally higher in countries with a high Human Development Index (HDI) (World Health Organization, 2017). Established risk factors include age, endogenous estrogen exposure, postmenopausal hormone therapy, nulliparity, smoking, alcohol consumption, family or personal history of breast cancer, increased breast density, and ionizing radiation. However, many cases still occur in women without known risk factors (World Health Organization, 2017). Despite substantial advances in treatment, important limitations remain, including restricted eligibility for some therapies and the emergence of drug resistance, underscoring the need for deeper mechanistic research (Wil et al., 2023).

In vertebrates, the TNNT family members expressed in striated muscle comprise three homologous variants: slow skeletal muscle TNNT (*TNNT1*), cardiac TNNT (*TNNT2*), and fast skeletal muscle TNNT (*TNNT3*) (Wei and Jin, 2016). Both *TNNT1* and *TNNT2* have been shown to be associated with various cardiac and skeletal muscle diseases, whereas the relationship between *TNNT3* and disease remains insufficiently studied. Given the central role of contractility-associated systems in cancer cell behavior and tumor mechanobiology, this relative gap in knowledge raises the question of whether *TNNT3* may also function outside striated muscle, including in pathological contexts such as breast cancer.

## 2 Breast cancer mechanobiology and the contractility-adhesion axis

The initiation, progression, and metastasis of breast cancer are not determined solely by genetic mutations, but matrix stiffness, solid stress, and interstitial fluid pressure within the tumor microenvironment also play important roles. For example, collagen crosslinking and fibrosis increase extracellular matrix stiffness, while tumor growth and vascular/lymphatic deformation elevate solid stress and interstitial fluid pressure. These mechanical cues regulate cells through pathways such as integrins, Rho GTPases, and Hippo–YAP/TAZ, thereby altering cancer-cell proliferation, invasion, stemness, and drug resistance (Zanconato et al., 2016). Targeting this process has become a common direction in basic, translational, and clinical research on breast cancer. Notably, multiple studies have shown that actomyosin contractility is one of the key factors driving cancer-cell metastasis (Aung et al., 2014). YAP/TAZ plays a regulatory role in stiffness sensing, and cancer-associated fibroblasts (CAFs) are directly associated with invasive, stem-like, and drug-resistant phenotypes across breast cancer subtypes, suggesting a link between extracellular stress and nuclear transcription and providing a solid bridge between myofilament contractility and tumor genes (Piccolo et al., 2023).

A core feature of the tumor mechanical microenvironment is a vicious cycle between increased matrix stiffness and enhanced cellular contractility. Matrix stiffness is a key mechanical factor driving malignant phenotypes in breast cancer, and cellular sensing of stiffness necessarily depends on the contractile apparatus. Changes in extracellular matrix composition, such as excessive collagen deposition and enhanced crosslinking, can promote cell growth and survival (Butcher et al., 2009). Extracellular matrix stiffness disrupts tissue morphogenesis by increasing cellular tension (Lo et al., 2000). Mouse models show that the stroma adjacent to transformed cells is considerably stiff, whereas normal extracellular matrix can promote the restoration of normal tissue architecture (Paszek et al., 2005). Together, these observations indicate that increased stiffness is an important factor in malignant progression.

## 3 *TNNT3* biology that may be relevant to non-muscle mechanotransduction

### 3.1 Canonical *TNNT3* biology and structural features

As a key component of the Tn complex, TnT3, together with troponin I (TNNI) and troponin C (TNNC), regulates muscle contraction; it has been shown to be associated with distal arthrogryposis, congenital myopathies, and other disorders, and has become a current research focus.

*TNNT3* is located at 11p15.5 and consists of 19 exons, of which exons 4–8 form an N-terminal variable region that undergoes alternative splicing, while exons 16 and 17 are mutually exclusive exons that encode a C-terminal variable segment (Jadidi et al., 2025). *TNNT3* exhibits complex alternative splicing. The *TNNT3* gene comprises multiple exons, with exons 4–8 in the N-terminal variable region undergoing alternative splicing and showing differential expression across developmental stages, thereby generating multiple protein isoforms (Stefancsik et al., 2003). These isoforms may modulate the thin filament response to Ca^2+^ by altering components of the Tn complex, such as troponin I (TnI) and troponin C (TnC), and their interactions with Tm, thereby affecting Ca^2+^ sensitivity and mechanical output.

**FIGURE 1:**
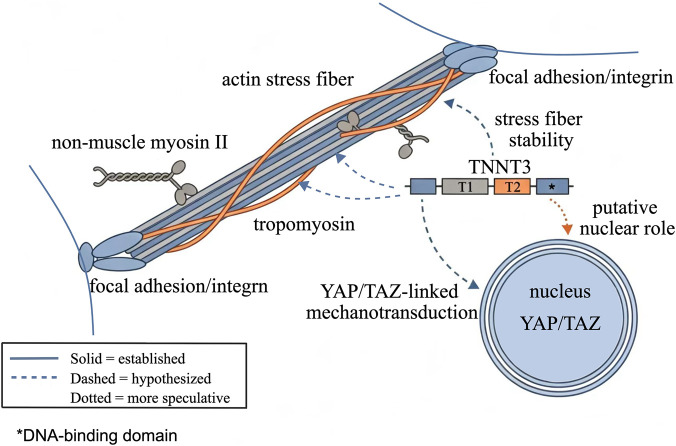
Structural organization of *TNNT3* and proposed interfaces with non-muscle contractility modules.

*TNNT3* encodes troponin T (TnT), the core structural subunit of the Tn complex, which also includes the Ca^2+^-binding subunit TnC and the inhibitory subunit TnI (Farah and Reinach, 1995). Functionally, TnT can be divided into two key segments that mediate interactions with distinct components on the thin filament. The T1 segment spans the proximal N-terminus to the middle region of TnT and anchors TnT to tropomyosin (Tm) on the thin filament (Heeley et al., 1987). The T2 segment is located at the C-terminus of TnT, interacts directly with TnC and TnI, and serves as a signaling hub that transmits Ca^2+^ signals and inhibitory signals to the thin filament (Heeley et al., 1987). These canonical structural features and the proposed non-muscle interfaces of *TNNT3* are summarized in Figure 1.

*TNNT3* contains a canonical T1 domain associated with Tm binding and a T2 domain associated with Tn-related regulatory interactions, together with a C-terminal region that has been linked to possible nuclear localization and a putative chromatin-associated role. In the simplified non-muscle context shown here, *TNNT3* is presented as a candidate interface with Tm-associated stress-fiber regulation, focal-adhesion/integrin-linked mechanotransduction, and downstream YAP/TAZ-associated signaling.

### 3.2 Why *TNNT3* has been considered conceptually relevant to non-muscle mechanotransduction

These structural and regulatory properties suggest that *TNNT3* may have the potential to influence contractile activity. Even in non-muscle cells lacking a fully organized sarcomeric architecture, *TNNT3* may still have the potential to influence contractility-related processes. The core of sarcomere contraction involves a series of conformational changes triggered by Ca^2+^ signaling, transmitted by Tn, accompanied by Tm displacement, and ultimately executed by myosin binding and force generation. When motor neuron signals trigger Ca^2+^ release from the sarcoplasmic reticulum, Ca^2+^ binds to the N-terminal domain of TnC and induces conformational rearrangement (Gordon et al., 2000). TnT is considered to connect the Tn–Tm–actin complex during sarcomeric contraction. Studies have shown that TnT and TnI form an asymmetric coiled-coil α-helix, the IT arm, approximately 80 Å in length, with its two ends connecting to and stabilizing two key Tm-binding sites on the thin filament (Takeda et al., 2003). Dysfunction of TnT has been demonstrated to be associated with multiple diseases: *TNNT1* is linked to nemaline myopathy, *TNNT2* to cardiomyopathies such as hypertrophic cardiomyopathy and dilated cardiomyopathy, and *TNNT3* has been preliminarily implicated in Sheldon–Hall syndrome and distal joint deformities (Wei and Jin, 2016).

**FIGURE 2:**
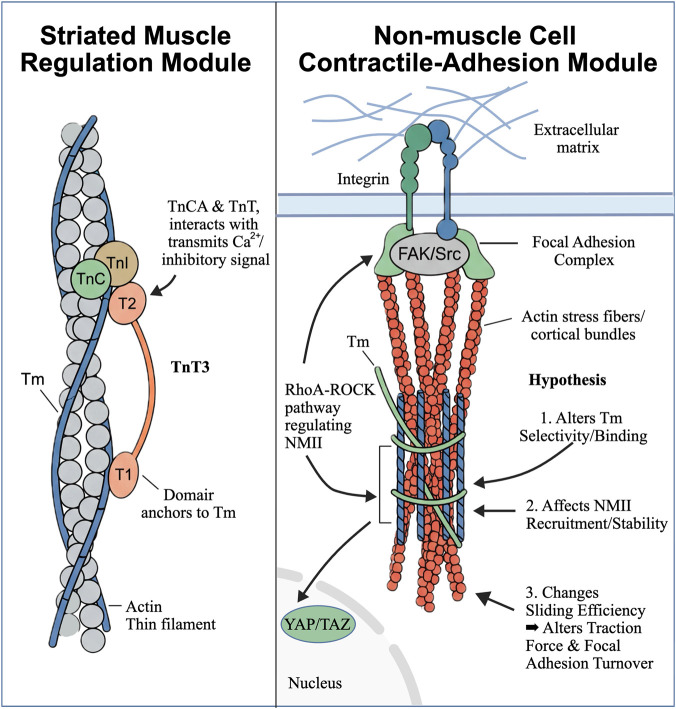
Proposed *TNNT3*-associated entry points into breast cancer mechanobiology.

The high conservation of contractile mechanisms between non-muscle and muscle cells provides a molecular basis for potential *TNNT3* involvement. Non-muscle cells are organized by stress fibers containing serially arranged, sarcomere-like contractile units whose bundle length, spacing, and stiffness are highly plastic (Livne and Geiger, 2016; Weirich et al., 2021). Using a new FRAP model, Takumi Saito and colleagues found that non-muscle cells can undergo remodeling within minutes to adapt to the microenvironment (Saito et al., 2022). In both non-muscle and muscle cells, F-actin serves as the structural scaffold, and myosin II provides the core source of force, with contraction relying on the cooperative activity of myosin on an actin-based framework. Studies have shown that, in both cell types, myosin II functions in the form of bipolar myosin filaments: these are typically smaller minifilaments in non-muscle cells and larger thick filaments in skeletal muscle, but the basic principle by which they drive contraction is identical (Chinthalapudi and Heissler, 2024). Moreover, myosin filaments drive the bound actin filaments toward their barbed ends, causing relative sliding between two sets of actin filaments and thereby generating contractile force (Dasanayake et al., 2011). In addition, both systems depend on a relatively conserved set of molecular components—including various actin-binding and crosslinking proteins such as α-actinin and Tm—that collectively regulate the structure and mechanical properties of F-actin networks or bundles, efficiently converting molecular-scale sliding into force output at the cellular and even tissue scale (Katsuta et al., 2023; Otey and Carpen, 2004). Thus, the potential relevance of *TNNT3* should not be considered strictly limited to sarcomeric contexts (Figure 2).

Importantly, these parallels do not constitute direct evidence that *TNNT3* is functionally incorporated into the non-muscle contractility machinery of breast cancer cells or stromal cells; rather, they provide the biological rationale for considering such a possibility.

In striated muscle, TnT3 anchors to Tm (T1 domain) and cooperates with TnC/TnI (T2 domain) to transmit Ca^2+^ and inhibitory signals, thereby controlling thin-filament activation. One hypothetical interface is that *TNNT3*, if present in a compatible non-muscle context, could interact with specific tropomyosin-associated modules and thereby influence actomyosin output.

### 3.3 Minimal molecular requirements for a putative *TNNT3* function outside striated muscle

We acknowledge that the question of what minimal molecular machinery is required for *TNNT3* to exert a functional effect outside striated muscle has not yet been resolved. However, in its canonical setting, *TNNT3* functions as part of a Tn–Tm regulatory assembly, in which TnT, TnI, and TnC cooperate on the thin filament; moreover, the C-terminal region of TnT is critical for TnI/TnC binding and Ca^2+^ sensitivity, whereas the N-terminal and central regions contribute to Tm anchoring (Jha et al., 1996). At the same time, TnT itself contains Tm-binding sites, suggesting that TnT3 might retain limited anchoring or modulatory capacity even in the absence of a fully reconstituted canonical Tn switch, although such a state would not be expected to recreate normal Ca^2+^-dependent thin-filament regulation (Amarasinghe and Jin, 2015). Importantly, experiments show that non-striated contexts are not necessarily devoid of Tn-related components: fast skeletal TnT, TnI, and TnC have been detected in vascular smooth muscle and shown to colocalize with Tm, indicating that partial or even broader reuse of Tn-linked modules can occur outside classic sarcomeres (Mo et al., 2008). In addition, *TNNT3* may have non-canonical functions that do not depend on full Tn-complex reconstitution. Nonmyofilament-associated TnT3 can enter the nucleus through a defined nuclear localization sequence, contains a leucine zipper DNA-binding domain, and can regulate nuclear localization of Cavβ1a through direct interaction (Zhang et al., 2015; Zhang et al., 2013). Taken together, we consider it unlikely that any putative *TNNT3* function in breast-cancer-related non-muscle cells would require complete reconstitution of a canonical striated-muscle TnT–TnI–TnC system. Rather, if *TNNT3* is functionally relevant, a more plausible minimal model would involve either partial reuse of an actin–Tm-associated module or non-canonical interactions, including nuclear trafficking and transcription-related signaling. In this framework, co-expression of TnI and TnC would strengthen the plausibility of a canonical-like mechanism, but should not be assumed to be obligatory for all *TNNT3*-associated effects. Accordingly, if *TNNT3* does not act through a fully reconstituted TnT–TnI–TnC complex in non-muscle cells, the best-supported alternative partners would most plausibly include specific Tm isoforms, given the intrinsic Tm-binding capacity of TnT. More speculative possibilities include other actin-associated proteins that organize thin-filament-like or stress-fiber-associated structures, as well as non-canonical interactors involved in nuclear trafficking or transcription-related signaling.

### 3.4 Non-canonical nuclear and chromatin-associated hypotheses for *TNNT3*

#### 3.4.1 Nuclear *TNNT3* as a putative chromatin-associated regulator

Previous work has shown that TnT3 contains a defined nuclear localization sequence in its C-terminal region, a possible nuclear export sequence, and a conserved leucine zipper DNA-binding domain, indicating that its nuclear trafficking is actively regulated rather than incidental (Zhang et al., 2013). Disruption of the nuclear localization sequence or the leucine zipper domain reduces TnT3-associated cytotoxicity, supporting the idea that nuclear localization and DNA-associated signaling are functionally relevant (Zhang et al., 2013). In addition, ChIP-seq analysis indicated that TnT3 can associate with a DNA consensus sequence containing the TGCCT motif, which overlaps with p53-binding elements, and enrichment analysis identified the p53 pathway as a prominent transcriptional context (Nunez Lopez et al., 2018). This possibility is further supported by the finding that the distal-arthrogryposis-linked p. R63C variant prolongs TnT3 half-life and promotes nuclear accumulation, suggesting that relatively small changes in protein stability could alter the size and persistence of the nuclear *TNNT3* pool (Lu et al., 2021). Accordingly, nuclear *TNNT3* is best viewed at present not as a proven transcription factor in breast cancer, but as a plausible chromatin-associated regulator whose effects, if present, are likely to depend on regulated nuclear trafficking, DNA-associated binding capacity, and protein stability.

#### 3.4.2 Locus-level chromatin regulation at 11p15.5

More broadly, a locus-level nuclear mechanism need not be restricted to direct DNA binding by *TNNT3* itself, but may also involve variant-dependent reconfiguration of local chromatin contacts and enhancer–promoter communication. Because tumor mechanical phenotypes depend on the coordinated regulation of focal-adhesion dynamics, the Rho-GTPase–ROCK–MLC axis, actin remodeling, and calcium signaling, variants at this locus could, in principle, influence tumor mechanics indirectly by altering the expression of other mechanobiology-relevant genes, even if *TNNT3* itself is only weakly expressed in tumor cells. The conceptual basis for this model lies in three-dimensional chromatin organization, in which topologically associating domains and chromatin loops bring distal enhancers into contact with target promoters. Variants that alter CTCF binding or cohesin-dependent loop architecture could therefore reconfigure enhancer–promoter pairing and shift local transcriptional programs (Nora et al., 2017; Rao et al., 2017; Lupiáñez et al., 2015). This hypothesis could be tested by generating three-dimensional interaction maps in breast-cancer-relevant cell types, integrating these data with chromatin accessibility, enhancer activity, expression quantitative trait locus (eQTL) or splicing quantitative trait locus (sQTL), and allele-specific chromatin signals, and then functionally perturbing candidate enhancers by CRISPR-based inhibition or activation. If such perturbations reproducibly alter both mechanobiology-related gene expression and phenotypes such as focal-adhesion dynamics, traction-force output, YAP/TAZ nuclear localization, or three-dimensional invasion, this would support the idea that the locus may influence tumor mechanical behavior through three-dimensional genome regulation rather than through *TNNT3* alone.

## 4 Current evidence linking *TNNT3* to breast cancer

To distinguish direct breast-cancer-related evidence from broader indirect support and explicitly speculative models, the current evidence framework relevant to TNNT3 is summarized in Table 1, with additional study-level details provided in Supplementary Table S1.

**TABLE 1:** Direct evidence, indirect support, and speculative models regarding TNNT3 in breast cancer mechanobiology.

Claim or topic	Main observation	Limitation	Confidence
Directly supported			
Positional candidate at 11p15.5	Within the 11p15.5 risk region Close to LSP1 and TNNI2	LD confounding Proximity ≠ causality	+ +
Low and inconsistent transcript-levels detectability	Detectable in breast-related datasets Subtype results inconsistent	No unified re-analysis Bulk dilution likely	+
Weak protein-level support	Near detection limit in most breast tumors Not high-abundance or subtype-defining	Underdetection due to low abundance/PTM/fragmentation not excluded	+ +
Indirectly suggestive			
Conceptual relevance to non-muscle mechanotransduction	Canonical Tm/TnI/TnC-related features Shared contractile logic across systems	No direct evidence shown in breast cancer cells/cafs/immune cells	+
Speculative			
Nuclear/chromatin-associated role	Nuclear localization Leucine zipper; p53-overlapping motif association	Not established in breast cancer	±

Confidence level: + +, moderate support; +, low support; ±, highly speculative.

### 4.1 Positional and expression-based observations relevant to *TNNT3*

*TNNT3* resides at 11p15.5, a breast cancer susceptibility region that also contains the established risk locus *LSP1* and the closely linked fast skeletal Tn gene *TNNI2*. *TNNT3* and *TNNI2* have long been recognized as closely linked within the fast skeletal Tn gene cluster at 11p15.5 (Barton et al., 1997). Independent genome-wide association studies first identified the *LSP1* region as a breast cancer susceptibility locus (Easton et al., 2007). More recent chromatin-interaction analyses at the 11p15.5-rs3817198 locus have revealed multiple interaction peaks, underscoring the regulatory complexity of this region and reinforcing the need for caution in assigning causality to any single nearby gene (Baxter et al., 2018). Accordingly, although *TNNT3* remains a plausible positional and biological candidate, the current evidence does not distinguish it from neighboring genes such as *LSP1* and *TNNI2*.

*TNNT3* is generally detectable in transcriptomic and proteomic assays, but its overall expression level is low and it does not exhibit broadly high abundance or consistently strong positive signals. Transcriptomic data from the Cancer Genome Atlas-Breast Invasive Carcinoma cohort (TCGA-BRCA) indicate that, although *TNNT3* can be detected in normal breast tissue, its expression remains at a low level (National Cancer Institute, 2020). Public-platform analyses showed a broadly similar subtype-associated tendency for TNNT3 expression, although the magnitude and statistical strength of the differences were not fully concordant, likely owing to differences in preprocessing, normalization, and subtype grouping strategy. To further clarify the platform-dependent variability, we re-analyzed TCGA-BRCA raw RNA-seq count data from the GDC portal using intrinsic subtype annotation from cBioPortal and a unified DESeq2 workflow. In this harmonized analysis, TNNT3 expression was higher in Basal-like than in HER2-enriched tumors (log2FC = 1.09, adjusted P = 5.99 × 10^-4). Nevertheless, one study combining TCGA expression validation with tumor-bearing mouse experiments observed by Western blot that TnT3 in TNBC tumor tissue can be manipulated and shows corresponding changes (Jia et al., 2025).

Although direct functional validation of *TNNT3* splice isoforms or adjacent non-coding transcripts in breast cancer is still lacking, studies have shown that, in muscle systems, *TNNT3* not only participates in contraction within myofibrils but also enters the nucleus and forms enrichment at specific genomic loci; its consensus sequence, TGCCT (CT)AG, overlaps with the p53-binding motif, suggesting that *TNNT3* may be associated with nuclear chromatin, which provides a potential direction (Nunez Lopez et al., 2018). In addition, it has been reported that, through bioinformatic analyses, genetic testing, and animal models, *TNNT3* is a potential drug target in triple-negative breast cancer (TNBC), although this conclusion still requires validation in independent populations and in cell-type–specific eQTL datasets (Jia et al., 2025).

### 4.2 Causal uncertainty at 11p15.5: *TNNT3*, *LSP1*, *TNNI2*, and other local candidates

The 11p15.5 signal should currently be interpreted within a framework of causal uncertainty rather than being assigned to *TNNT3* alone. *TNNT3* remains a plausible candidate because it lies within the risk region, is positioned close to *LSP1* and *TNNI2* in a dense linkage disequilibrium (LD) block, and is mechanistically attractive in light of its putative relevance to contractility-associated biology, nuclear localization, DNA motif association, and p53-overlapping binding. However, these features primarily support biological plausibility rather than locus-specific causality, particularly because *TNNT3* expression is generally low and inconsistent across breast-related datasets and direct functional validation in breast cancer is still lacking. By contrast, *LSP1* currently has stronger prior breast-relevant support, as large studies have linked the locus, including rs3817198, to mammographic density and breast cancer risk, although this does not establish *LSP1* as the sole causal effector in such an LD-dense region (Easton et al., 2007). *TNNI2* also remains a plausible candidate gene due to its positional proximity, reported nuclear localization, and direct interaction with ERRα (Li et al., 2008). Furthermore, a *TNNI2*/ERRα/SIRT1 axis has been associated with altered tumor cell behavior in pancreatic cancer (Fu et al., 2021). However, there is little direct breast cancer–specific evidence linking the 11p15.5 signal to *TNNI2*. In addition, the broader 11p15.5 interval contains other biologically relevant regulatory candidates, including H19, IGF2, and 91H, all of which underscore the complexity of this imprinted and regulatory domain (Table 2). Therefore, future studies should combine statistical fine-mapping with cell-type-specific eQTL and sQTL colocalization analyses to determine whether the associated signal preferentially maps to TNNT3, LSP1, TNNI2, or other nearby transcripts. In parallel, allele-specific expression analysis in isogenic cell lines carrying edited risk and non-risk haplotypes could clarify which local gene is most directly responsive to the regulatory variant. CRISPRi/a perturbation of candidate enhancers across the locus, followed by parallel assessment of TNNT3, LSP1, and TNNI2 expression and function, would further help assign enhancer-to-gene relationships. Finally, chromatin interaction mapping and spatially resolved expression analyses would be important to determine whether the relevant regulatory effects occur in tumor cells, stromal fibroblasts, immune cells, or other focal cellular contexts.

**TABLE 2:** Comparison of TNNT3 and alternative candidate genes at the 11p15.5 breast cancer risk locus.

Candidate gene	What supports candidacy	What limits interpretation	Evidence still needed
*TNNT3*	Positional candidate Mechanobiology relevance Nuclear/chromatin plausibility	Low/inconsistent expression No direct breast-cancer validation Plausibility ≠ causality	Fine-mapping Cell-type-specific eQTL/sQTL Parallel perturbation
*LSP1*	Stronger breast-risk Mammographic-density evidence	No sole causality from epidemiology LD confounding remains	Enhancer–promoter assignment Comparative perturbation
*TNNI2*	Positional candidate Nuclear localization ERRα co-activator precedent	Little breast-specific locus evidence No direct colocalization support	Breast-relevant expression/QTL/contact mapping Functional testing
Other local regulatory (e.g., *H19/IGF2/91H*)	Imprinted-domain complexity Established breast-cancer relevance	Signal-specific assignment unresolved Imprinting findings inconsistent	Locus-wide chromatin mapping Coding/non-coding parallel perturbation

## 5 Mechanistic hypotheses and future directions

**FIGURE 3:**
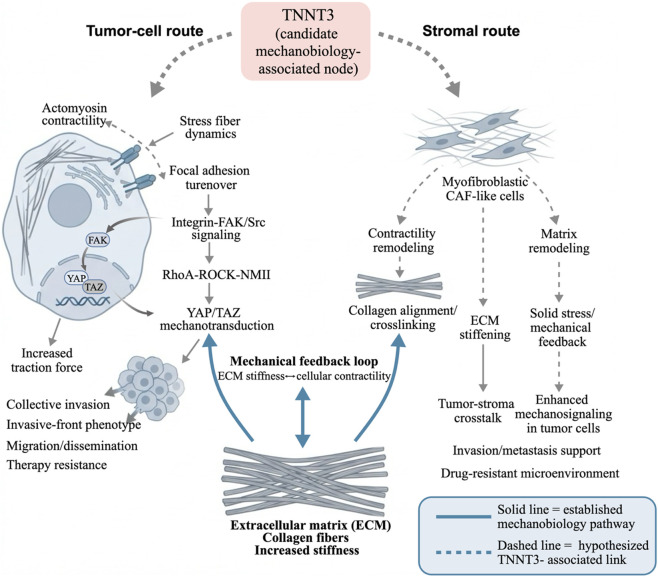
Hypothetical spatial and cellular contexts for *TNNT3*-associated effects in breast cancer.

As a canonical regulator of contraction, *TNNT3*, if heterogeneously expressed in breast cancer cells or cells within the microenvironment, may directly engage the relevant mechanical feedback loop by modulating contractile modules in non-muscle cells. Critically, after cells sense stiffness *via* integrins, they rely on intracellular contractile force to promote focal-adhesion maturation and activate FAK/Src signaling (Dupont et al., 2011). Studies have shown that inhibiting lysyl oxidase (LOX)–mediated collagen crosslinking reduces matrix fibrosis and stiffness, concomitantly weakens focal adhesion and integrin-associated signaling, thereby suppressing invasive phenotypes and delaying tumor progression (Levental et al., 2009). Therefore, molecules that may affect the integrity of the cellular contractile apparatus, including *TNNT3*, could potentially influence the stiffness-response threshold by altering contractile output. In the tumor microenvironment, if *TNNT3* is upregulated in cancer-associated fibroblasts (CAFs) or invasive breast cancer cells, it may influence non-muscle contractility and focal-adhesion dynamics through shared Tm-associated modules (Figure 3). Noticeably, any proposed TNNT3-associated mechanism should be interpreted in the context of the currently weak expression- and protein-level support in breast cancer. Accordingly, the models discussed below are intended as hypothesis-generating possibilities rather than evidence-supported explanations.

*TNNT3* is shown as a candidate mechanobiology-associated node that may intersect with breast cancer progression through putative tumor-cell and stromal routes. In tumor cells, *TNNT3*-associated effects, if present, may converge on contractility, focal-adhesion signaling, the RhoA–ROCK–NMII axis, and YAP/TAZ-linked mechanotransduction, with potential consequences for invasion, dissemination, and therapy resistance. In stromal contexts, *TNNT3*-associated effects may involve myofibroblastic CAF-like cells, matrix remodeling, extracellular matrix stiffening, and tumor–stroma mechanical crosstalk. Solid lines denote established mechanobiology pathways, and dashed lines denote hypothesized *TNNT3*-associated links.

### 5.1 Putative tumor-cell route: contractility and collective invasion

This model remains tentative, particularly because current bulk transcriptomic and proteomic data do not support a broad or abundant *TNNT3* signal in breast tumor cells. During breast cancer progression, tumor cells undergo pronounced remodeling of mechanical phenotypes, one prominent feature of which involves epithelial–mesenchymal transition and collective-migration–associated changes in tissue behavior. *In vitro* 3D culture studies have shown that prominent leader cells can be readily identified at the front of invasive chains in tumor organoids (Cheung et al., 2013). This indicates that breast cancer often infiltrates surrounding tissues *via* collective migration rather than single-cell migration, in which a small number of functionally specialized cells are positioned at the invasive front, maintain cell–cell contacts, and guide the subsequent cells forward. Friedl has provided a detailed explanation of this process (Friedl et al., 2012). Three-dimensional cell culture models further suggest that spatial sorting may occur within tumor cell collectives during collective invasion: differences in cell–cell or cell–matrix adhesion properties, together with differences in intracellular actomyosin contractile forces, may jointly drive more invasive cells to become enriched at the matrix interface or the invasive front, thereby shaping specific invasive structures (Devanny et al., 2021). Within three-dimensional matrices, tumor-cell invasion depends on the cooperation between mechanical forces and protease-mediated degradation. In a 3D Matrigel model, when local compressive–traction stress increases with enhanced mechanical confinement and reaches an approximate threshold of 165 Pa, the invasion mode shifts from a deformation-based, protease-independent pattern to a protease-dependent program accompanied by the formation of invadopodia-like protrusions, MT1-MMP enrichment, and increased degradative activity (Aung et al., 2014). This finding indicates that, in a confined three-dimensional microenvironment, elevated local compressive stress serves as an important mechanical cue that triggers cells to initiate an MT1-MMP–centered degradative invasion program to breach the matrix barrier.

If *TNNT3* is expressed in a focal subset of invasive-front tumor cells lacking a typical sarcomeric structure, any associated effect would more likely reflect partial reuse of contractility-related modules or non-canonical thin-filament interactions rather than the canonical troponin-based mode seen in striated muscle. Given the conserved nature of contractile mechanisms, any *TNNT3*-associated effect in breast cancer cells, if present, might influence adhesion maturation or migration speed by altering stress-fiber-associated contractility. More specific effects on NMII recruitment, binding stability, or sliding rate remain hypothetical. Because Tm isoforms in non-muscle cells shape NMII recruitment and tension output, one hypothetical consequence would be altered coupling between Ca^2+^-associated signals and actomyosin-generated tension. This possibility remains untested in breast cancer. Such effects could, in principle, influence stress-fiber homeostasis, focal-adhesion dynamics, or migration speed. Under this model, *TNNT3*-associated effects might secondarily converge on traction-force generation and YAP/TAZ-linked mechanosignaling, although no direct *TNNT3*-dependent mechanism has yet been established. To minimize technical bias arising from antibody cross-reactivity and signal admixture from stromal cells, a multi-layered orthogonal validation framework should be established, including spatial co-localization analysis, isoform-specific transcript validation, and protein detection supported by targeted mass spectrometry or gene-knockout controls. On this basis, one testable prediction is that *TNNT3* overexpression, knockdown, or binding-defective mutations would be expected to induce systematic changes in single-cell traction-force magnitude and anisotropy, focal-adhesion dynamics, and FAK/Src signaling activity. If these phenotypes are markedly attenuated by ROCK or NMII inhibition, or show selective dependence on Ca^2+^-related perturbations, this would further support the idea that *TNNT3* participates in tumor-cell mechanical regulation through contractility-associated pathways.

### 5.2 Putative stromal route: CAF-associated matrix stiffening and tumor-stroma feedback

Given the weak support for broad epithelial *TNNT3* expression, a stromal or niche-restricted model should be considered only as one possible alternative explanation rather than as a preferred resolution. An alternative possibility is that *TNNT3*-associated loci may influence the tumor mechanical microenvironment indirectly through stromal pathways. CAFs exhibit marked heterogeneity, and myofibroblast-like subsets typically display stronger actomyosin contractility, maintain higher tissue tension, and promote extracellular matrix stiffening. In multiple models, these changes are accompanied by upregulation of crosslinking-related factors (Erdogan et al., 2017). Such a high-tension and high-stiffness microenvironment may promote signaling through integrin-linked focal adhesions, FAK/Src, and the Rho-ROCK axis. It may also influence mechanosensitive transcriptional programs such as YAP/TAZ, with potential consequences for tumor-cell migration, invasion, and therapeutic response (Zhang and Zhang, 2025).

If *TNNT3*-locus effects act primarily in stromal cells, they may favor CAF phenotypes with greater contractility and matrix-remodeling capacity. This, in turn, could alter tissue stiffness and stress distribution without requiring high *TNNT3* expression in tumor epithelial cells. Under this model, *TNNT3*-locus variation would be expected to influence stromal contractility, matrix remodeling, and tumor–stroma mechanical crosstalk, ultimately altering tumor-cell mechanosensing and invasive behavior. This mechanism could be systematically assessed using genotype-stratified single-cell transcriptomics and spatial omics together with concurrent measurements of tissue mechanical parameters, and further tested through CAF-specific genetic or epigenetic perturbation.

### 5.3 More speculative routes: immune-cell mechanophenotypes and paracrine signaling

These routes are even more speculative and are presented here primarily to outline testable alternatives under conditions of weak bulk-level support. Paracrine communication mediated by exosomes or extracellular vesicles may represent one possible route for transmitting stromal- or tumor-associated mechanical phenotypes. Such vesicles can carry RNAs and proteins capable of influencing cytoskeletal organization and adhesion signaling in recipient cells. However, there is currently no direct evidence that *TNNT3*, or *TNNT3*-associated regulatory products, is selectively packaged and functionally delivered through this route (Skog et al., 2008; Sung et al., 2021). This possibility is therefore better regarded as a secondary and more speculative mechanism rather than a central model.

A more plausible alternative is that variants near the *TNNT3* locus may influence the immune microenvironment through cell-type-specific regulatory effects in immune cells rather than through broad *TNNT3* expression in tumor epithelial cells. Such effects may also interact with extracellular matrix stiffness and tissue mechanics. Such effects could alter pathways governing mechanosensing, cytoskeletal organization, adhesion, and migration, thereby affecting immune-cell infiltration, positioning, and tissue penetration under different physical conditions (Nicolas-Boluda et al., 2021; Rømer et al., 2021). Under this model, tumors carrying the risk allele might show differences in immune infiltration, spatial distribution, and functional-state markers. They might also show altered spatial relationships between immune features and local mechanical properties, such as collagen alignment, matrix densification, adhesion signaling, or mechanosensitive transcriptional programs. At the single-cell level, such effects might also appear as allele-dependent differences in mechanically relevant gene expression within immune cells, potentially accompanied by measurable changes in migration, adhesion, or immune-synapse mechanics (Mei et al., 2024; Friedman-et al., 2024; Ritter et al., 2015). These possibilities could be evaluated using genotype-stratified single-cell transcriptomics and spatial-omics analyses combined with multiplex immune phenotyping, matrix imaging, and tissue mechanics measurements.

### 5.4 Threshold- and state-dependent models for low-level *TNNT3* activity

We agree that, if *TNNT3* is expressed at very low levels in breast-cancer-related contexts, any associated effect is unlikely to scale linearly with bulk abundance and may instead be threshold- and state-dependent. This possibility is biologically plausible because *TNNT3* itself appears to be mechanoresponsive. In C2C12 cells, mechanical stretch alters *TNNT3* pre-mRNA alternative splicing through a cell-autonomous, Akt-dependent mechanism, and *TNNT3* splicing is quantitatively regulated by body weight and external load in rats (Schilder et al., 2012; Schilder et al., 2011). In addition, as discussed above, TNNT3 may have non-canonical nuclear properties. This leaves open the possibility that small changes in TNNT3 localization or stability could be amplified through nuclear mechanisms.

A threshold-dependent interpretation is also compatible with the downstream YAP/TAZ axis, although the available literature does not directly establish a *TNNT3*-dependent mechanism. Experiments show that force can drive YAP nuclear entry by mechanically regulating transport across nuclear pores (Elosegui-Artola et al., 2017). In addition, YAP/TAZ activity varies across the cell cycle with a reported peak in G1 (Kim et al., 2019). YAP-dependent transcription has also been shown to depend on dynamic localization-reset behavior rather than on static nuclear abundance alone (Franklin et al., 2020). These observations suggest that the YAP/TAZ axis is context dependent and not fully captured by a simple linear relationship with static nuclear abundance.

Collectively, these observations support a model in which any *TNNT3*-associated effect, if present in breast cancer, may become detectable only under conditions of sufficiently high mechanical stress, actomyosin–nucleus coupling, or specific cell-state windows, rather than as a uniform effect across all tumor cells.

### 5.5 *TNNT3* as a candidate biomarker rather than a prioritized therapeutic target

Although *TNNT3* has been discussed here as a candidate mechanobiology-associated node, its current evidence base is not sufficient to prioritize it over broader mechanotransduction regulators such as ROCK, YAP/TAZ, or FAK as a therapeutic target. These established nodes occupy more central positions in the broader network linking contractility, adhesion, and mechanotransduction and are therefore more likely to produce stronger and more generalizable effects when perturbed. By contrast, any *TNNT3*-associated role in breast cancer, if validated, may prove to be spatially restricted, cell-type-specific, and limited to particular mechanical states. Under this framework, the main potential advantage of *TNNT3* would not necessarily be greater efficacy, but greater contextual specificity. This could make *TNNT3* more relevant as a candidate biomarker of restricted stromal or invasive-front states, or as a mechanistically informative locus-linked candidate, rather than as a broadly actionable therapeutic target. At present, however, even this more limited translational potential remains hypothetical and requires direct spatial, functional, and cell-type-resolved validation.

## 6 Limitations, alternative explanations and key knowledge gaps

### 6.1 Interpretive constraints

Despite the genetic and mechanobiological rationale discussed above, the current evidence linking *TNNT3* to breast cancer remains limited and must be interpreted in light of several observations that argue against a broad or dominant role, including low bulk-level expression, weak or near-undetectable protein-level support in most breast tumor cohorts, and the absence of strong proteogenomic evidence for *TNNT3* as a high-abundance, subtype-defining, or prognostically robust molecule. These negative or weak-support observations should therefore be treated as central interpretive constraints rather than as minor caveats. Another major uncertainty is that the primary effect of the 11p15.5 risk variants may not be mediated by sustained *TNNT3* expression within tumor cells, but may instead arise indirectly through regulation of neighboring genes or broader network nodes. Because risk signals are often located in multi-gene regulatory regions and LD structure may implicate nearby genes by proximity alone, causal-gene assignment remains difficult. In addition, colocalization results are influenced by population background, tissue sources, sample size, and multifactorial architecture. Accordingly, neighboring genes should be considered as alternative explanations with equal weight, and integrated evidence combining fine mapping, allele-specific expression, chromatin interactions, and functional perturbation will be required for more definitive inference. A further limitation of the harmonized subtype re-analysis is that no clearly suitable batch variable was available for formal batch correction in the accessible annotation tables. An exploratory sensitivity analysis adjusting for tissue source site attenuated the TNNT3 subtype signal, suggesting that source-site heterogeneity may contribute to the observed subtype contrast.

In the vast majority of breast tumor epithelia, *TNNT3* is negative or near the detection limit, and large-cohort omics analyses have not shown stable prognostic value or a clear association with specific histological subtypes, grade, or stage (Mertins et al., 2016). Multiple large-scale proteomics studies, including integrated genomic analyses of CPTAC breast cancer proteome-related datasets, have not reported *TNNT3* as a high-abundance protein in breast cancer epithelial cells, a subtype-defining feature, or a prognostically relevant molecule (Krug et al., 2020). Pan-cancer proteomic atlases likewise have not listed *TNNT3* as a potential tumor marker (Knol et al., 2025).

### 6.2 Possible explanations for weak TNNT3 detectability

One possible explanation for the very weak *TNNT3* protein signal in breast tissue is that *TNNT3* may be subject to rapid turnover or post-translational regulation that is not well captured by routine untargeted proteomics. This possibility is biologically plausible because *TNNT3* has been reported to undergo ubiquitin–proteasome-dependent degradation, and a disease-linked p. R63C variant can prolong its half-life and promote nuclear accumulation (Lu et al., 2021). In addition, TnT3 can exist not only as full-length protein but also as proteolytic fragments, since age-associated TnT3 fragmentation and C-terminal fragment accumulation have been observed in skeletal muscle. The same study showed that calpain inhibition reduces TnT3 fragmentation and restores downstream functional readouts, supporting the idea that proteolytic processing can substantially reshape the detectable *TNNT3* species pool without simply reflecting full-length abundance (Zhang et al., 2016). More broadly, prior work on Tn proteins has shown that phosphorylation can alter susceptibility to calpain-mediated degradation, raising the possibility that post-translational modification could further influence *TNNT3* detectability, although this has not yet been directly demonstrated in breast cancer (Di Lisa et al., 1995). These explanations remain hypothetical in breast cancer, and low expression may simply indicate limited biological relevance in many settings. Nevertheless, they suggest that weak protein-level support should be interpreted cautiously rather than taken as definitive evidence against any possible context-dependent role. For this reason, standard bulk proteomics may underestimate *TNNT3* if the relevant species are low-abundance, unstable, highly modified, or present predominantly as fragments rather than intact protein.

To test this possibility directly, methods optimized for low-abundance, unstable, and post-translationally regulated protein species will be needed. At the protein level, targeted mass spectrometry, especially SRM, MRM, or PRM, would be more suitable than routine untargeted proteomics for detecting low-level *TNNT3* peptides, particularly when combined with prior immunoprecipitation or other enrichment strategies. To assess protein stability, cycloheximide chase experiments together with proteasome inhibition and calpain inhibition could be used to determine whether *TNNT3* undergoes rapid degradation or proteolytic fragmentation in relevant breast cancer models. To examine post-translational regulation, immunoprecipitation followed by anti-ubiquitin immunoblotting, phosphopeptide enrichment coupled with LC MS/MS, and ubiquitin remnant profiling could help identify ubiquitination-associated or phosphorylation-associated *TNNT3* species. In parallel, proximity ligation assay and *in situ* proximity ligation assay may be useful for detecting low-abundance *TNNT3* and its modification-associated or partner-associated signals in cells or tissue sections, especially when conventional immunohistochemistry lacks sufficient sensitivity. Because *TNNT3* also shows complex alternative splicing, isoform-specific RT qPCR, long-read RNA sequencing, and proteoform-aware mass spectrometric approaches would further help distinguish whether the very weak bulk signal reflects true absence, rapid turnover, extensive modification, or the predominance of specific fragments or splice-dependent species.

## 7 Conclusion

*TNNT3* remains a biologically interesting but currently low-evidence candidate at the intersection of breast cancer genetics and mechanobiology. Existing data do not establish a direct or generalizable role for *TNNT3* in breast cancer progression, but leave open the possibility of a spatially restricted, cell-type-specific, or context-dependent function. At present, its relevance should be regarded as hypothesis-generating rather than demonstrated. Further spatial, cell-type-specific, and functional studies will be required to determine whether *TNNT3* has any reproducible mechanistic or translational significance in breast cancer.
