# Supplementary material for: TNNT3 as a candidate node in breast cancer mechanobiology: current evidence, mechanistic models, and key knowledge gaps
Source: Front Cell Dev Biol. 2026 May 15;14:1836170. doi: 10.3389/fcell.2026.1836170 (PMC13219267; doi:10.3389/fcell.2026.1836170)
Supplement: Supplementary file 1 [file DataSheet1.pdf]

**Supplementary Table S1. Summary of breast-cancer-related evidence directly relevant to TNNT3**

| Study type | Model/system | Breast-cancer context | Readout | Main finding | Key limitation | Confidence |
|---|---|---|---|---|---|---|
| Positional/locus-based evidence[1, 2] | Human breast-cancer risk-locus studies | 11p15.5 susceptibility region | GWAS locus mapping; chromatin interaction context | TNNT3 retained as a positional candidate; located near LSP1 and TNNI2 | Positional support only; dense LD; no gene-specific causal resolution | ++ |
| Transcript-level observation[3-5] | TCGA-BRCA; UALCAN; GEPIA2 | Breast tissue; subtype comparison | Bulk RNA expression; subtype stratification | Low-level TNNT3 expression in BRCA | Low-level signal; non-identical subtype definitions across platforms. | + |
| TNBC-focused integrative evidence[6] | Summary-data genetics with downstream validation; tumor-bearing mouse context | Triple-negative breast cancer | SMR; colocalization; transcript/protein validation | TNNT3 proposed as a TNBC-related candidate target; tumor-tissue protein change reported under treatment conditions | Indirect target nomination; no direct TNNT3 perturbation; treatment-response context | + |
| Breast proteogenomic evidence[7, 8] | Primary human breast tumors | Breast-cancer proteomic landscape | Mass spectrometry-based proteogenomics | No strong TNNT3-centered positive signal reported in major breast proteogenomic findings | Untargeted proteomics; weak-support / absence-of-signal evidence; not a TNNT3-targeted assay | ++ |

| | | | | | | |
|---|---|---|---|---|---|---|
| Pan-cancer proteomic evidence including breast tumors[9] | Multi-cancer human tumor proteome datasets | Biomarker / tumor-marker context | Mass spectrometry-based atlas analysis | TNNT3 not highlighted as a major tumor biomarker signal | Pan-cancer rather than breast-specific; absence-of-signal evidence only | ++ |

**Confidence level:** + +, moderate support; +, low support

## References


1. D. F. Easton, K. A. Pooley, A. M. Dunning, P. D. Pharoah, D. Thompson, D. G. Ballinger, J. P. Struewing, J. Morrison, H. Field, R. Luben, N. Wareham, S. Ahmed, C. S. Healey, R. Bowman, K. B. Meyer, C. A. Haiman, L. K. Kolonel, B. E. Henderson, L. Le Marchand, P. Brennan, S. Sangrajrang, V. Gaborieau, F. Odefrey, C. Y. Shen, P. E. Wu, H. C. Wang, D. Eccles, D. G. Evans, J. Peto, O. Fletcher, N. Johnson, S. Seal, M. R. Stratton, N. Rahman, G. Chenevix-Trench, S. E. Bojesen, B. G. Nordestgaard, C. K. Axelsson, M. Garcia-Closas, L. Brinton, S. Chanock, J. Lissowska, B. Peplonska, H. Nevanlinna, R. Fagerholm, H. Eerola, D. Kang, K. Y. Yoo, D. Y. Noh, S. H. Ahn, D. J. Hunter, S. E. Hankinson, D. G. Cox, P. Hall, S. Wedren, J. Liu, Y. L. Low, N. Bogdanova, P. Schürmann, T. Dörk, R. A. Tollenaar, C. E. Jacobi, P. Devilee, J. G. Klijn, A. J. Sigurdson, M. M. Doody, B. H. Alexander, J. Zhang, A. Cox, I. W. Brock, G. MacPherson, M. W. Reed, F. J. Couch, E. L. Goode, J. E. Olson, H. Meijers-Heijboer, A. van den Ouweland, A. Uitterlinden, F. Rivadeneira, R. L. Milne, G. Ribas, A. Gonzalez-Neira, J. Benitez, J. L. Hopper, M. McCredie, M. Southey, G. G. Giles, C. Schroen, C. Justenhoven, H. Brauch, U. Hamann, Y. D. Ko, A. B. Spurdle, J. Beesley, X. Chen, A. Mannermaa, V. M. Kosma, V. Kataja, J. Hartikainen, N. E. Day, D. R. Cox and B. A. Ponder, Genome-wide association study identifies novel breast cancer susceptibility loci. Nature 447, 1087-1093 (2007)
2. P. J. Barton, P. J. Townsend, N. J. Brand and M. H. Yacoub, Localization of the fast skeletal muscle troponin I gene (TNNI2) to 11p15.5: genes for troponin I and T are organized in pairs. Ann Hum Genet 61, 519-523 (1997)
3. National Cancer Institute, in The Cancer Genome Atlas Program (TCGA). [Internet]. https://www.cancer.gov/ccg/research/genome-sequencing/tcga Accessed 15 February 2026
4. D. S. Chandrashekar, S. K. Karthikeyan, P. K. Korla, H. Patel, A. R. Shovon, M. Athar, G. J. Netto, Z. S. Qin, S. Kumar, U. Manne, C. J. Creighton and S. Varambally, UALCAN: An update to the integrated cancer data analysis platform. Neoplasia 25, 18-27 (2022)
5. Z. Tang, B. Kang, C. Li, T. Chen and Z. Zhang, GEPIA2: an enhanced web server for large-scale expression profiling and interactive analysis Nucleic Acids Research 47(W1), W556–W560 (2019)

6. J. Jia, Y. Ma, D. Wu, Y. Dou, J. Yuan, F. Liu and A. Shi, Integrated summary data-based Mendelian randomization and colocalization analysis reveals TNNT3 as a target of Deer-Antler-Ginseng-formula for triple-negative breast cancer. Discover Oncology 16, 2022 (2025)
7. P. Mertins, D. Mani, K. V. Ruggles, M. A. Gillette, K. R. Clauser, P. Wang, X. Wang, J. W. Qiao, S. Cao and F. Petralia, Proteogenomics connects somatic mutations to signalling in breast cancer. Nature 534, 55-62 (2016)
8. K. Krug, E. J. Jaehnig, S. Satpathy, L. Blumenberg, A. Karpova, M. Anurag, G. Miles, P. Mertins, Y. Geffen and L. C. Tang, Proteogenomic landscape of breast cancer tumorigenesis and targeted therapy. Cell 183, 1436-1456. e1431 (2020)
9. J. C. Knol, M. Lyu, F. Böttger, M. N. Monteiro, T. V. Pham, F. Rolfs, A. Vallés-Martí, T. Schelfhorst, R. R. de Goeij-de Haas and I. V. Bijnsdorp, The pan-cancer proteome atlas, a mass spectrometry-based landscape for discovering tumor biology, biomarkers, and therapeutic targets. Cancer Cell 43, 1328-1346. e1328 (2025)
